# Marasmus—Opium

**Published:** 1897-03

**Authors:** E. V. Ross

**Affiliations:** Rochester, N. Y.


### MARASMUS—OPIUM.

E. V. Ross, M. D., Rochester, N. Y.

August 22d, was called at two A. M. to see a child who was thought to be dying, and, on arriving at the house, I found the most marasmic specimen of humanity that has been my fortune to see. A male child æt. ten months. Bottle fed. (Condensed milk diluted.) Sick two weeks, under regular (?) treatment.

*Status præsens.* Appearance that of a little dried-up old man, emaciated in the extreme. Rolls the head. Eyes half open. Pupils contracted to the size of a pin's head. Eyes turned upward. Lower jaw dropped down. Appears stupid. Stools watery, dark, and offensive.

I did not give a very hopeful prognosis; to this the father replied: "Well, Doctor, I don't believe that a great deal can be done, but do the best you can." With this I took out Bell on *Diarrhœa* and my interleaved copy of Lippe's *Repertory* and went to work. After one-half hour's study I worked out the following:

*Looks old.* Ant-crud., Argt-nit., Arsen., Calc-carb., *Calc-phos.*, Cinchon., Hepar, *Iod.*, Mag-carb., Natr-carb., *Natr-mur.*, Nux-vom., *Op.*, Phos., Podo., Rheum, *Sarsap.*, Sep., *Sulph.*, Sulph-ac.

*Emaciation.* Ambr., *Amm-c.*, Anac., *Ant.*, Apis, Arn., *Ars.*, *Bar-c.*, Bell., Borax, Bry., *Calc.*, Carbo-veg., Clem., Cham., *Chin.*, Cina, Cocc., Cupr., Dig., Dros., Dulc., *Ferr.*, *Graph.*, Guaj., Hep., Ign., *Iod.*, Ipec., Kali-bich., Lach., *Lyc.*, Magn-c., Merc., Mez., Natr-c., *Natr-mur.*, *Nitr-ac.*, Nux-m., *Nux-v.*, Op., *Petr.*, Phos., *Phos-ac.*, Plb., *Puls.*, Raph., Samb., *Sass.*, *Sec-c.*, Selen., Sep., Sil., *Stann.*, Staph., Stront., *Sulph.*, *Tabac.*, Thuj., Verat.

*Rolling of head.* *Bell.*, Bry., Cicuta, Colch., *Hell.*, Hyos., Kali-brom., *Podo.*, (Sec.) Sil., Stram., Verat-alb., (Verat-vir.) Zinc.

*Eyes half open.* Bell., Ferr-phos., Hell., *Op.*, Podo., Sulph.

*Pupils contracted.* Anac., Ars., Bell., Camph., Cham., Chelid., Cicuta, Cocc., Croton., Daph., Dros., Hæmotox., Ign., Lach., Mang., Merc-corr., Mez., Nux-m., Nux-v., *Op.*, Plb., Puls., Rheum, Samb., Scilla., Sec-c., Sep., Sil., Sulph., Thuja, Verat-alb., Zinc.

— — in cholera infantum. Cycl., *Op.*, *Verat-alb.*

*Eyes turned upward.* Apis, Cicuta, Cupr-nit., Hell., Mosch., *Op.*

*Dropping of lower jaw.* Arn., Ars., Carbo-veg., Hell., Kali-iod., *Lach.*, Lyc., *Op.*, Stram., Variola.

— — — — with stupor. Lyc., *Op.*, Sulph.

*Stupor.* Ang., *Ant.*, Ars., *Asaf.*, *Bar-c.*, Bell., Bry., Camph., *Caust.*, Coloc., Con., *Croc.*, Cupr., *Dig.*, *Laur.*, *Led.*, *Nux-m.*, Nux-v., *Op.*, Phos., *Phos-ac.*, *Plb.*, Puls., *Sec-c.*, Sep., *Stram.*, Tart., Tereb., *Verat.*, *Zinc.*, Zing.

Dark, watery, offensive stool is met by many remedies, including Opium.

From the above it is readily seen, without further analysis, that Opium is *the* remedy. It was therefore administered as follows: ℞ *Opium*$^{200}$ (Dunham) Chart. XII, Sig; powder every two hours until better or dead (for it could not be worse). Diet: Egg albumen and water.

August 23d, 10 A. M.—Child greatly improved. Gave placebo and ordered fresh milk, one-half pint; water, one-half pint; cream, four tablespoonfuls; Peptogen Milk Powder, one measure.

I would state, of all the infant foods Peptogen Milk Powder has given me the best results.* I ordered a Seibert steam sterilizer, the contents of a No. 1 (3 oz.) bottle, to be fed every two hours. It should be borne in mind that the *weight*, not the age, of the infant determines the food properly, for this purpose it is quite convenient to have Seibert's table to refer to. Some objections may be raised to this method of feeding, on the grounds that Seibert based his conclusion on the relative size of the stomach, to the weight of the child *in a healthy, growing state;* this is theorectically correct, but practically I have observed it to work admirably in these marasmic conditions.

To be brief, the child continued to improve; a few days later it received *Sulphur*$^{55m}$, to remove a red excoriated condition about the anus.

It is well and gaining flesh rapidly to date, September 22d, 1896.

* *Vide* an article by R. H. Chittenden, Ph. D., *New York Medical Journal*, July 18th, 1896, p. 71.

